# 7-Chloro-4-oxo-4*H*-chromene-3-carbaldehyde

**DOI:** 10.1107/S1600536814014925

**Published:** 2014-07-02

**Authors:** Yoshinobu Ishikawa

**Affiliations:** a School of Pharmaceutical Sciences, University of Shizuoka, 52-1 Yada, Suruga-ku, Shizuoka 422-8526, Japan

**Keywords:** crystal structure

## Abstract

In the title compound, C_10_H_5_ClO_3_, a chlorinated 3-formyl­chromone derivative, all atoms are essentially coplanar (r.m.s. deviation = 0.0592 Å for all non-H atoms), with the largest deviation from the least-squares plane [0.1792 (19) Å] being for the chromone-ring carbonyl O atom. In the crystal, mol­ecules are linked through C—H⋯O hydrogen bonds to form tetrads, which are assembled by stacking inter­actions [centroid–centroid distance between the pyran rings = 3.823 (3) Å] and van der Waals contacts between the Cl atoms [Cl⋯Cl = 3.4483 (16) Å and C—Cl⋯Cl = 171.73 (7)°] into a three-dimensional architecture.

## Related literature   

For related structures, see: Ishikawa & Motohashi (2013 ▶); Ishikawa (2014*a* ▶,*b* ▶). For halogen bonding, see: Auffinger *et al.* (2004 ▶); Metrangolo *et al.* (2005 ▶); Wilcken *et al.* (2013 ▶); Sirimulla *et al.* (2013 ▶). For halogen–halogen inter­actions, see: Metrangolo & Resnati (2014 ▶); Mukherjee & Desiraju (2014 ▶).
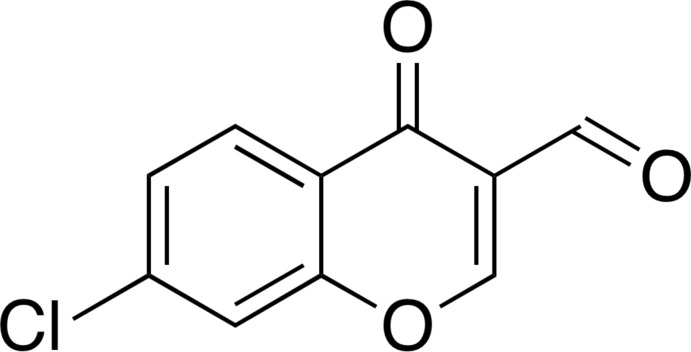


## Experimental   

### 

#### Crystal data  

C_10_H_5_ClO_3_
*M* *_r_* = 208.60
Triclinic, 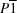

*a* = 3.823 (2) Å
*b* = 5.973 (3) Å
*c* = 18.386 (8) Å
α = 85.99 (4)°
β = 87.74 (4)°
γ = 86.58 (4)°
*V* = 417.8 (4) Å^3^
*Z* = 2
Mo *K*α radiation
μ = 0.43 mm^−1^
*T* = 100 K
0.42 × 0.25 × 0.08 mm

#### Data collection  

Rigaku AFC-7R diffractometer
Absorption correction: ψ scan (North *et al.*, 1968 ▶) *T*_min_ = 0.865, *T*_max_ = 0.966
2429 measured reflections
1899 independent reflections
1690 reflections with *F* ^2^ > 2σ(*F* ^2^)
*R*_int_ = 0.050
3 standard reflections every 150 reflections intensity decay: −1.1%

#### Refinement  

*R*[*F* ^2^ > 2σ(*F* ^2^)] = 0.037
*wR*(*F* ^2^) = 0.104
*S* = 1.10
1899 reflections
127 parameters
H-atom parameters constrained
Δρ_max_ = 0.41 e Å^−3^
Δρ_min_ = −0.50 e Å^−3^

### 

Data collection: *WinAFC Diffractometer Control Software* (Rigaku, 1999 ▶); cell refinement: *WinAFC Diffractometer Control Software*; data reduction: *WinAFC Diffractometer Control Software*; program(s) used to solve structure: *SIR2008* (Burla *et al.*, 2007 ▶); program(s) used to refine structure: *SHELXL97* (Sheldrick, 2008 ▶); molecular graphics: *CrystalStructure* (Rigaku, 2010 ▶); software used to prepare material for publication: *CrystalStructure*.

## Supplementary Material

Crystal structure: contains datablock(s) General, I. DOI: 10.1107/S1600536814014925/tk5323sup1.cif

Structure factors: contains datablock(s) I. DOI: 10.1107/S1600536814014925/tk5323Isup2.hkl

Supporting information file. DOI: 10.1107/S1600536814014925/tk5323Isup3.cml

CCDC reference: 1010095

Additional supporting information:  crystallographic information; 3D view; checkCIF report

## Figures and Tables

**Table 1:** Hydrogen-bond geometry (Å, °)

*D*—H⋯*A*	*D*—H	H⋯*A*	*D*⋯*A*	*D*—H⋯*A*
C7—H4⋯O2^i^	0.95	2.34	3.204 (3)	151 (1)
C1—H1⋯O3^ii^	0.95	2.37	3.209 (3)	148 (1)

Symmetry codes: (i) 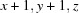; (ii) 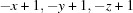.

**Fig. 1.**
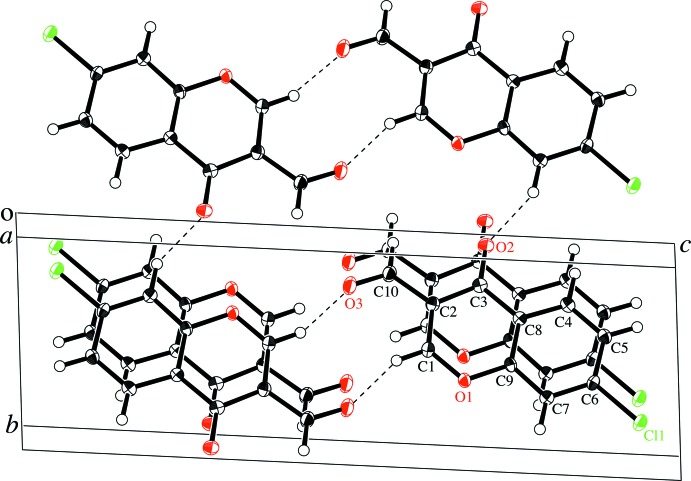
A packing view of the title compound, with displacement ellipsoids drawn at the 50% probability level. C—H···O hydrogen bonds are represented by dashed lines.

**Fig. 2.**
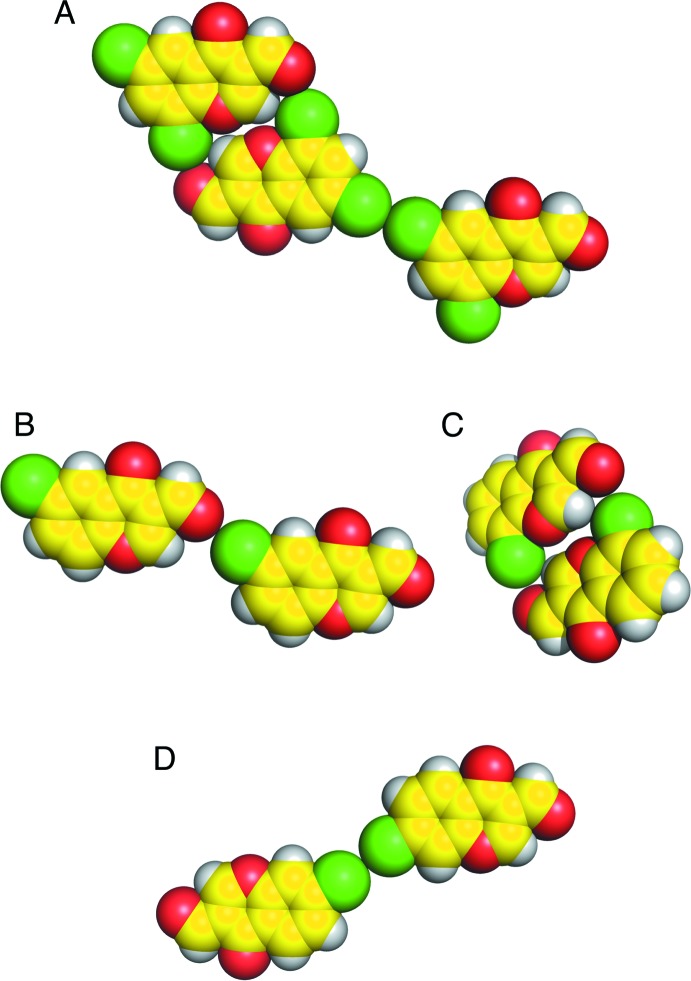
Sphere models of the crystal structures of 6,8-dichloro-4-oxochromene-3-carbaldehyde (A, Ishikawa & Motohashi, 2013), 6-chloro-4-oxo-4H-chromene-3-carbaldehyde (B, Ishikawa, 2014a), 8-chloro-4-oxo-4H-chromene-3-carbaldehyde (C, Ishikawa, 2014b), and the title compound (D).

## supplementary crystallographic information

### S1. Structural commentary

Halogen bonding and halogen···halogen inter­actions have recently attracted much attention in medicinal chemistry, chemical biology, supra­molecular chemistry and crystal engineering (Auffinger *et al.*, 2004, Metrangolo *et al*., 2005, Wilcken *et al.*, 2013, Sirimulla *et al.*, 2013, Mukherjee & Desiraju, 2014, Metrangolo & Resnati, 2014). We have recently reported the crystal structures of a dichlorinated 3-formyl­chromone derivative 6,8-di­chloro-4-oxochromene-3-carbaldehyde (Ishikawa & Motohashi, 2013), and monochlorinated 3-formyl­chromone derivatives 6-chloro-4-oxo-4*H*-chromene-3-carbaldehyde (Ishikawa, 2014*a*) and 8-chloro-4-oxo-4*H*-chromene-3-carbaldehyde (Ishikawa, 2014*b*). Halogen bonding between the formyl oxygen atom and the chlorine atom at 8-position and type I halogen···halogen inter­action between the chlorine atoms at 6-position are observed in 6,8-di­chloro-4-oxochromene-3-carbaldehyde (Fig·2 A). On the other hand, van der Waals contacts between the formyl oxygen atom and the chlorine atom at 6-position in 6-chloro-4-oxo-4*H*-chromene-3-carbaldehyde (Fig·2 B) and between the formyl oxygen atom and the chlorine atom at 8-position in 8-chloro-4-oxo-4*H*-chromene-3-carbaldehyde (Fig·2 C) are found. As part of our inter­est in these types of chemical bonding, we herein report the crystal structure of a monochlorinated 3-formyl­chromone derivative 7-chloro-4-oxo-4*H*-chromene-3-carbaldehyde. The objective of this study is to reveal whether a short contact is found for the chlorine atom at 7-position.

The mean deviation of the least-square planes for the non-hydrogen atoms is 0.0592 Å, and the largest deviation is 0.1792 (19) Å for O3.

In the crystal, the molecules are linked through C–H···O hydrogen bonds among the translation-symmetry^i^ and inversion-symmetry equivalents^ii,iii^ to form tetrads [i: *x* – 1, *y* – 1, *z*, ii: –*x*, –*y*, –*z* + 1, iii: –*x* + 1, –*y* + 1, –*z* + 1], which are assembled by stacking inter­actions [centroid–centroid distance between the pyran rings = 3.823 (3) Å], as shown in Fig. 1.

Van der Waals contacts between the chlorine atoms of inversion-symmetry equivalents are found [Cl1···Cl1^iv^ = 3.4483 (16) Å, C6–Cl1···Cl1^iv^ = 171.73 (7)°, iv: –*x* + 1, –*y* + 2, –*z* + 2], as shown in Fig. 2D. Thus, significant short contact for the chlorine atom at 7-position is not observed. Whereas the characteristic short Cl···O contact is observed in the dichlorinated 3-formyl­chromone (Fig. 2A), such a short contact is not found in the monochlorinated ones (Fig. 2B, C and D). These findings should be helpful to understand inter­actions of halogenated ligands with proteins, and thus invaluable for rational drug design.

### S2. Synthesis and crystallization

To a solution of 4-chloro-2-hy­droxy­aceto­phenone (5.9 mmol) in *N*,*N*-di­methyl­formamide (15 ml) was added dropwise POCl_3_ (14.7 mmol) at 0 °C. After the mixture was stirred for 14 h at room temperature, water (50 ml) was added. The precipitates were collected, washed with water, and dried *in vacuo* (yield: 85%). ^1^H NMR (400 MHz, CDCl_3_): *δ* = 7.48 (d, 1H, *J* = 8.8 Hz), 7.57 (s, 1H), 8.24 (d, 1H, *J* = 8.8 Hz), 8.52 (s, 1H), 10.37 (s, 1H). DART-MS calcd for [C_10_H_5_Cl_1_O_3_ + H^+^]: 209.001, found 209.029. Single crystals suitable for X-ray diffraction were obtained from a 1,2-di­chloro­ethane/cyclo­hexane solution of the title compound at room temperature.

### S3. Refinement

Crystal data, data collection and structure refinement details are summarized in Table 1. The C(*sp*^2^)-bound hydrogen atoms were placed in geometrical positions [C—H 0.95 Å, *U*_iso_(H) = 1.2*U*_eq_(C)], and refined using a riding model.

### Crystal data

**Table:**

C_10_H_5_ClO_3_	*Z* = 2
*M**_r_* = 208.60	*F*(000) = 212.00
Triclinic, *P*1	*D*_x_ = 1.658 Mg m^−^^3^
Hall symbol: -P 1	Mo *K*α radiation, λ = 0.71069 Å
*a* = 3.823 (2) Å	Cell parameters from 25 reflections
*b* = 5.973 (3) Å	θ = 15.2–17.0°
*c* = 18.386 (8) Å	µ = 0.43 mm^−^^1^
α = 85.99 (4)°	*T* = 100 K
β = 87.74 (4)°	Plate, yellow
γ = 86.58 (4)°	0.42 × 0.25 × 0.08 mm
*V* = 417.8 (4) Å^3^	

### Data collection

**Table:**

Rigaku AFC-7R diffractometer	*R*_int_ = 0.050
ω–2θ scans	θ_max_ = 27.5°
Absorption correction: ψ scan (North *et al.*, 1968)	*h* = −4→2
*T*_min_ = 0.865, *T*_max_ = 0.966	*k* = −7→7
2429 measured reflections	*l* = −23→23
1899 independent reflections	3 standard reflections every 150 reflections
1690 reflections with *F*^2^ > 2σ(*F*^2^)	intensity decay: −1.1%

### Refinement

**Table:**

Refinement on *F*^2^	Secondary atom site location: difference Fourier map
*R*[*F*^2^ > 2σ(*F*^2^)] = 0.037	Hydrogen site location: inferred from neighbouring sites
*wR*(*F*^2^) = 0.104	H-atom parameters constrained
*S* = 1.10	*w* = 1/[σ^2^(*F*_o_^2^) + (0.0425*P*)^2^ + 0.5019*P*] where *P* = (*F*_o_^2^ + 2*F*_c_^2^)/3
1899 reflections	(Δ/σ)_max_ = 0.001
127 parameters	Δρ_max_ = 0.41 e Å^−^^3^
0 restraints	Δρ_min_ = −0.50 e Å^−^^3^
Primary atom site location: structure-invariant direct methods	

### Special details

**Table:**

Refinement. Refinement was performed using all reflections. The weighted *R*-factor (*wR*) and goodness of fit (*S*) are based on *F*^2^. *R*-factor (gt) are based on *F*. The threshold expression of *F*^2^ > 2.0 σ(*F*^2^) is used only for calculating *R*-factor (gt).

### Fractional atomic coordinates and isotropic or equivalent isotropic displacement parameters (Å^2^)

**Table:**

	*x*	*y*	*z*	*U*_iso_*/*U*_eq_	
Cl1	0.40101 (12)	0.80466 (8)	0.94032 (2)	0.01990 (15)	
O1	0.3743 (4)	0.6471 (3)	0.67507 (7)	0.0171 (3)	
O2	−0.1431 (4)	0.0675 (3)	0.71337 (8)	0.0199 (3)	
O3	0.2057 (5)	0.2458 (3)	0.50473 (8)	0.0265 (4)	
C1	0.3077 (5)	0.5184 (4)	0.62091 (10)	0.0162 (4)	
C2	0.1464 (5)	0.3212 (4)	0.62998 (10)	0.0160 (4)	
C3	0.0256 (5)	0.2365 (3)	0.70234 (10)	0.0152 (4)	
C4	0.0441 (5)	0.3071 (4)	0.83526 (10)	0.0159 (4)	
C5	0.1313 (5)	0.4379 (4)	0.88996 (10)	0.0164 (4)	
C6	0.2909 (5)	0.6400 (3)	0.87083 (10)	0.0150 (4)	
C7	0.3697 (5)	0.7116 (3)	0.79955 (10)	0.0153 (4)	
C8	0.1182 (5)	0.3730 (3)	0.76188 (10)	0.0138 (4)	
C9	0.2831 (5)	0.5741 (3)	0.74571 (10)	0.0140 (4)	
C10	0.0936 (6)	0.1938 (4)	0.56551 (11)	0.0201 (4)	
H1	0.3784	0.5687	0.5727	0.0194*	
H2	−0.0674	0.1709	0.8474	0.0191*	
H3	0.0840	0.3919	0.9397	0.0197*	
H4	0.4785	0.8490	0.7877	0.0183*	
H5	−0.0367	0.0629	0.5723	0.0241*	

### Atomic displacement parameters (Å^2^)

**Table:**

	*U*^11^	*U*^22^	*U*^33^	*U*^12^	*U*^13^	*U*^23^
Cl1	0.0221 (3)	0.0227 (3)	0.0159 (3)	−0.00304 (17)	−0.00206 (16)	−0.00573 (17)
O1	0.0201 (7)	0.0185 (7)	0.0129 (7)	−0.0055 (5)	0.0019 (5)	−0.0007 (5)
O2	0.0204 (7)	0.0195 (7)	0.0207 (8)	−0.0079 (6)	0.0003 (6)	−0.0018 (6)
O3	0.0359 (9)	0.0303 (9)	0.0146 (8)	−0.0111 (7)	0.0031 (6)	−0.0054 (6)
C1	0.0151 (9)	0.0198 (9)	0.0139 (9)	−0.0017 (7)	−0.0004 (7)	−0.0019 (7)
C2	0.0145 (9)	0.0181 (9)	0.0154 (9)	−0.0010 (7)	−0.0012 (7)	−0.0018 (7)
C3	0.0108 (8)	0.0168 (9)	0.0181 (9)	−0.0001 (7)	−0.0010 (7)	−0.0015 (7)
C4	0.0122 (9)	0.0182 (9)	0.0171 (9)	−0.0010 (7)	0.0001 (7)	0.0011 (7)
C5	0.0158 (9)	0.0179 (9)	0.0151 (9)	0.0001 (7)	0.0010 (7)	0.0007 (7)
C6	0.0134 (9)	0.0181 (9)	0.0138 (9)	0.0002 (7)	−0.0021 (7)	−0.0042 (7)
C7	0.0126 (9)	0.0167 (9)	0.0167 (9)	−0.0017 (7)	−0.0004 (7)	−0.0016 (7)
C8	0.0109 (8)	0.0152 (9)	0.0151 (9)	−0.0000 (6)	0.0005 (7)	−0.0010 (7)
C9	0.0121 (8)	0.0173 (9)	0.0122 (9)	−0.0005 (7)	0.0004 (6)	0.0007 (7)
C10	0.0225 (10)	0.0232 (10)	0.0154 (10)	−0.0048 (8)	−0.0013 (7)	−0.0036 (8)

### Geometric parameters (Å, º)

**Table:**

Cl1—C6	1.745 (2)	C4—C8	1.402 (3)
O1—C1	1.341 (3)	C5—C6	1.402 (3)
O1—C9	1.379 (3)	C6—C7	1.377 (3)
O2—C3	1.231 (3)	C7—C9	1.392 (3)
O3—C10	1.208 (3)	C8—C9	1.398 (3)
C1—C2	1.359 (3)	C1—H1	0.950
C2—C3	1.458 (3)	C4—H2	0.950
C2—C10	1.480 (3)	C5—H3	0.950
C3—C8	1.476 (3)	C7—H4	0.950
C4—C5	1.378 (3)	C10—H5	0.950
			
O1···C3	2.865 (3)	C10···H1	2.5619
O1···C6	3.598 (3)	H1···H5	3.4961
O2···C1	3.577 (3)	H2···H3	2.3320
O2···C4	2.877 (3)	Cl1···H2^iii^	3.1871
O2···C10	2.895 (3)	Cl1···H2^iv^	3.4009
O3···C1	2.827 (3)	Cl1···H3^ii^	3.4824
C1···C7	3.578 (3)	Cl1···H3^xi^	3.0426
C1···C8	2.759 (3)	Cl1···H3^xii^	3.1343
C2···C9	2.777 (3)	O1···H5^iii^	3.3638
C4···C7	2.809 (3)	O2···H4^v^	2.3412
C5···C9	2.769 (3)	O2···H4^vi^	2.9752
C6···C8	2.769 (3)	O3···H1^ix^	2.8238
Cl1···Cl1^i^	3.4483 (16)	O3···H1^x^	2.3652
Cl1···C5^ii^	3.578 (3)	O3···H5^ii^	3.2929
O1···O2^iii^	3.202 (3)	O3···H5^viii^	2.5304
O1···O2^iv^	3.333 (3)	C1···H5^iii^	3.5044
O1···C2^ii^	3.540 (3)	C2···H1^vii^	3.3800
O1···C3^ii^	3.415 (3)	C2···H5^ii^	3.5566
O1···C8^ii^	3.571 (3)	C3···H4^v^	3.4647
O2···O1^v^	3.333 (3)	C3···H4^vi^	3.1692
O2···O1^vi^	3.202 (3)	C4···H2^ii^	3.4575
O2···C2^vii^	3.397 (3)	C4···H4^vi^	3.2692
O2···C3^vii^	3.286 (3)	C5···H2^ii^	3.4558
O2···C7^v^	3.204 (3)	C5···H3^xi^	3.4146
O2···C7^vi^	3.185 (3)	C6···H2^iii^	3.3840
O2···C8^vii^	3.393 (3)	C6···H3^ii^	3.5349
O2···C9^vi^	3.309 (3)	C7···H2^iii^	3.2808
O3···O3^viii^	3.430 (3)	C7···H4^vii^	3.4681
O3···O3^ix^	3.332 (3)	C8···H4^vi^	3.3537
O3···C1^ix^	3.278 (3)	C9···H4^vii^	3.4840
O3···C1^x^	3.209 (3)	C10···H1^vii^	3.4338
O3···C10^viii^	3.289 (3)	C10···H1^ix^	3.3580
C1···O3^ix^	3.278 (3)	C10···H1^x^	3.4611
C1···O3^x^	3.209 (3)	C10···H5^ii^	3.3751
C1···C2^ii^	3.356 (3)	C10···H5^viii^	3.0735
C1···C3^ii^	3.468 (3)	H1···O3^ix^	2.8238
C2···O1^vii^	3.540 (3)	H1···O3^x^	2.3652
C2···O2^ii^	3.397 (3)	H1···C2^ii^	3.3800
C2···C1^vii^	3.356 (3)	H1···C10^ii^	3.4338
C3···O1^vii^	3.415 (3)	H1···C10^ix^	3.3580
C3···O2^ii^	3.286 (3)	H1···C10^x^	3.4611
C3···C1^vii^	3.468 (3)	H1···H1^x^	2.9506
C3···C9^vii^	3.481 (3)	H1···H5^iii^	3.2659
C4···C6^vii^	3.467 (3)	H1···H5^ix^	3.5735
C4···C7^vii^	3.476 (3)	H2···Cl1^v^	3.4009
C5···Cl1^vii^	3.578 (3)	H2···Cl1^vi^	3.1871
C5···C6^vii^	3.386 (4)	H2···C4^vii^	3.4575
C6···C4^ii^	3.467 (3)	H2···C5^vii^	3.4558
C6···C5^ii^	3.386 (4)	H2···C6^vi^	3.3840
C7···O2^iii^	3.185 (3)	H2···C7^vi^	3.2808
C7···O2^iv^	3.204 (3)	H2···H4^v^	2.9597
C7···C4^ii^	3.476 (3)	H2···H4^vi^	2.9822
C7···C8^ii^	3.479 (3)	H3···Cl1^vii^	3.4824
C8···O1^vii^	3.571 (3)	H3···Cl1^xi^	3.0426
C8···O2^ii^	3.393 (3)	H3···Cl1^xii^	3.1343
C8···C7^vii^	3.479 (3)	H3···C5^xi^	3.4146
C8···C9^vii^	3.360 (3)	H3···C6^vii^	3.5349
C9···O2^iii^	3.309 (3)	H3···H3^xi^	2.6802
C9···C3^ii^	3.481 (3)	H4···O2^iii^	2.9752
C9···C8^ii^	3.360 (3)	H4···O2^iv^	2.3412
C10···O3^viii^	3.289 (3)	H4···C3^iii^	3.1692
C10···C10^viii^	3.575 (4)	H4···C3^iv^	3.4647
Cl1···H3	2.8121	H4···C4^iii^	3.2692
Cl1···H4	2.8064	H4···C7^ii^	3.4681
O1···H4	2.5238	H4···C8^iii^	3.3537
O2···H2	2.6135	H4···C9^ii^	3.4840
O2···H5	2.6106	H4···H2^iii^	2.9822
O3···H1	2.5045	H4···H2^iv^	2.9597
C1···H5	3.2854	H5···O1^vi^	3.3638
C3···H1	3.2928	H5···O3^vii^	3.2929
C3···H2	2.6818	H5···O3^viii^	2.5304
C3···H5	2.6956	H5···C1^vi^	3.5044
C5···H4	3.2981	H5···C2^vii^	3.5566
C6···H2	3.2536	H5···C10^vii^	3.3751
C7···H3	3.2895	H5···C10^viii^	3.0735
C8···H3	3.2766	H5···H1^vi^	3.2659
C8···H4	3.3034	H5···H1^ix^	3.5735
C9···H1	3.1896	H5···H5^viii^	2.8132
C9···H2	3.2637		
			
C1—O1—C9	118.62 (16)	C4—C8—C9	118.32 (18)
O1—C1—C2	124.73 (17)	O1—C9—C7	115.76 (17)
C1—C2—C3	120.42 (18)	O1—C9—C8	121.83 (18)
C1—C2—C10	119.24 (17)	C7—C9—C8	122.41 (17)
C3—C2—C10	120.34 (18)	O3—C10—C2	124.1 (2)
O2—C3—C2	123.38 (18)	O1—C1—H1	117.638
O2—C3—C8	122.38 (17)	C2—C1—H1	117.633
C2—C3—C8	114.24 (17)	C5—C4—H2	119.644
C5—C4—C8	120.70 (18)	C8—C4—H2	119.653
C4—C5—C6	118.78 (17)	C4—C5—H3	120.611
Cl1—C6—C5	118.56 (15)	C6—C5—H3	120.611
Cl1—C6—C7	118.76 (15)	C6—C7—H4	121.444
C5—C6—C7	122.66 (18)	C9—C7—H4	121.444
C6—C7—C9	117.11 (18)	O3—C10—H5	117.967
C3—C8—C4	121.70 (17)	C2—C10—H5	117.976
C3—C8—C9	119.98 (17)		
			
C1—O1—C9—C7	177.96 (14)	C8—C4—C5—C6	0.9 (3)
C1—O1—C9—C8	−1.3 (3)	C8—C4—C5—H3	−179.1
C9—O1—C1—C2	1.8 (3)	H2—C4—C5—C6	−179.1
C9—O1—C1—H1	−178.2	H2—C4—C5—H3	0.9
O1—C1—C2—C3	1.1 (3)	H2—C4—C8—C3	0.4
O1—C1—C2—C10	−179.09 (14)	H2—C4—C8—C9	−179.8
H1—C1—C2—C3	−178.9	C4—C5—C6—Cl1	−179.93 (14)
H1—C1—C2—C10	0.9	C4—C5—C6—C7	−1.1 (3)
C1—C2—C3—O2	174.85 (16)	H3—C5—C6—Cl1	0.1
C1—C2—C3—C8	−4.3 (3)	H3—C5—C6—C7	178.9
C1—C2—C10—O3	5.1 (3)	Cl1—C6—C7—C9	178.98 (11)
C1—C2—C10—H5	−174.9	Cl1—C6—C7—H4	−1.0
C3—C2—C10—O3	−175.13 (16)	C5—C6—C7—C9	0.2 (3)
C3—C2—C10—H5	4.9	C5—C6—C7—H4	−179.8
C10—C2—C3—O2	−4.9 (3)	C6—C7—C9—O1	−178.22 (14)
C10—C2—C3—C8	175.94 (14)	C6—C7—C9—C8	1.0 (3)
O2—C3—C8—C4	5.4 (3)	H4—C7—C9—O1	1.8
O2—C3—C8—C9	−174.38 (15)	H4—C7—C9—C8	−179.0
C2—C3—C8—C4	−175.44 (14)	C3—C8—C9—O1	−2.2 (3)
C2—C3—C8—C9	4.8 (3)	C3—C8—C9—C7	178.65 (14)
C5—C4—C8—C3	−179.64 (15)	C4—C8—C9—O1	178.01 (14)
C5—C4—C8—C9	0.1 (3)	C4—C8—C9—C7	−1.1 (3)

Symmetry codes: (i) −*x*+1, −*y*+2, −*z*+2; (ii) *x*+1, *y*, *z*; (iii) *x*, *y*+1, *z*; (iv) *x*+1, *y*+1, *z*; (v) *x*−1, *y*−1, *z*; (vi) *x*, *y*−1, *z*; (vii) *x*−1, *y*, *z*; (viii) −*x*, −*y*, −*z*+1; (ix) −*x*, −*y*+1, −*z*+1; (x) −*x*+1, −*y*+1, −*z*+1; (xi) −*x*, −*y*+1, −*z*+2; (xii) −*x*+1, −*y*+1, −*z*+2.

### Hydrogen-bond geometry (Å, º)

**Table:**

*D*—H···*A*	*D*—H	H···*A*	*D*···*A*	*D*—H···*A*
C7—H4···O2^iv^	0.95	2.34	3.204 (3)	151 (1)
C1—H1···O3^x^	0.95	2.37	3.209 (3)	148 (1)

Symmetry codes: (iv) *x*+1, *y*+1, *z*; (x) −*x*+1, −*y*+1, −*z*+1.

## References

[bb1] Auffinger, P., Hays, F. A., Westhof, E. & Ho, P. S. (2004). *Proc. Natl Acad. Sci. USA*, **101**, 16789–16794. 10.1073/pnas.0407607101 PMC529416 15557000

[bb2] Burla, M. C., Caliandro, R., Camalli, M., Carrozzini, B., Cascarano, G. L., De Caro, L., Giacovazzo, C., Polidori, G., Siliqi, D. & Spagna, R. (2007). *J. Appl. Cryst.* **40**, 609–613.

[bb3] Ishikawa, Y. (2014*a*). *Acta Cryst.* E**70**, o514. 10.1107/S1600536814007119 PMC4011265 24860329

[bb4] Ishikawa, Y. (2014*b*). *Acta Cryst.* E**70**, o743. 10.1107/S1600536814012483 PMC4120543 25161540

[bb5] Ishikawa, Y. & Motohashi, Y. (2013). *Acta Cryst.* E**69**, o1416. 10.1107/S1600536813022228 PMC3884433 24427051

[bb6] Metrangolo, P., Neukirch, H., Pilati, T. & Resnati, G. (2005). *Acc. Chem. Res.* **38**, 386–395. 10.1021/ar0400995 15895976

[bb7] Metrangolo, P. & Resnati, G. (2014). *IUCrJ*, **1**, 5–7. 10.1107/S205225251303491X PMC4104972 25075314

[bb8] Mukherjee, A. & Desiraju, G. R. (2014). *IUCrJ*, **1**, 49–60. 10.1107/S2052252513025657 PMC4104968 25075319

[bb9] North, A. C. T., Phillips, D. C. & Mathews, F. S. (1968). *Acta Cryst.* A**24**, 351–359.

[bb10] Rigaku (1999). *WinAFC Diffractometer Control Software* Rigaku Corporation, Tokyo, Japan.

[bb11] Rigaku (2010). *CrystalStructure* Rigaku Corporation, Tokyo, Japan.

[bb12] Sheldrick, G. M. (2008). *Acta Cryst.* A**64**, 112–122. 10.1107/S0108767307043930 18156677

[bb13] Sirimulla, S., Bailey, J. B., Vegesna, R. & Narayan, M. (2013). *J. Chem. Inf. Model.* **53**, 2781–2791. 10.1021/ci400257k 24134121

[bb14] Wilcken, R., Zimmermann, M. O., Lange, A., Joerger, A. C. & Boeckler, F. M. (2013). *J. Med. Chem.* **56**, 1363–1388. 10.1021/jm3012068 23145854

